# CPS1 augments hepatic glucagon response through CaMKII/FOXO1 pathway

**DOI:** 10.3389/fphar.2024.1437738

**Published:** 2024-08-13

**Authors:** Xiao-Meng Sun, Xin Wu, Meng-Guang Wei, Li-Zeng Zhu, Wen-hui Wu, Xin-Yue Zhou, Lian-Wen Qi, Qun Liu

**Affiliations:** ^1^ State Key Laboratory of Natural Medicines, School of Traditional Chinese Pharmacy, China Pharmaceutical University, Nanjing, China; ^2^ Clinical Metabolomics Center, China Pharmaceutical University, Nanjing, China

**Keywords:** type 2 diabetes, gluconeogenesis, carbamoyl phosphate synthetase 1, CaMKII/FOXO1 pathway, cynarin

## Abstract

**Introduction:** Elevated glucagon levels are a characteristic feature of type 2 diabetes. This abnormal increase in glucagon can lead to an accelerated rate of gluconeogenesis. Glucagon also stimulates hepatic metabolism of amino acids, particularly promoting the formation of urea. The specific role of carbamoyl phosphate synthetase 1 (CPS1), a rate-limiting enzyme in the urea cycle, in the development versus the persistence of glucagon-induced hyperglycemia has not been previously established.

**Methods:** The study employed both *in vivo* and *in vitro* approaches to assess the impact of CPS1 modulation on glucagon response. CPS1 was knockdown or overexpression to evaluate its influence on hepatic gluconeogenesis. In addition, an *in-silico* strategy was employed to identify a potential CPS1 inhibitor.

**Results:** Knockdown of CPS1 significantly reduced the glucagon response both *in vivo* and *in vitro*. Conversely, overexpression of CPS1 resulted in an overactive hepatic gluconeogenic response. Mechanistically, CPS1 induced the release of calcium ions from the endoplasmic reticulum, which in turn triggered the phosphorylation of CaMKII. The activation of CaMKII then facilitated the dephosphorylation and nuclear translocation of FOXO1, culminating in the enhancement of hepatic gluconeogenesis. Furthermore, cynarin, a natural CPS1 inhibitor derived from the artichoke plant, had the capacity to attenuate the hepatic glucagon response in a CPS1-dependent manner.

**Discussion:** CPS1 played a pivotal role in mediating glucagon-induced hepatic gluconeogenesis. The discovery of cynarin as a natural inhibitor of CPS1 suggested its potential as a therapeutic agent for diabetes treatment.

## 1 Introduction

Type 2 diabetes mellitus (T2DM) is intricately linked with a spectrum of metabolic disorders, including hyperlipidemia, non-alcoholic fatty liver disease, and obesity (Calcutt et al., 2009; Chen et al., 2019; Yu et al., 2019). Hyperglycemia, characterized by elevated blood glucose levels, is a defining clinical feature of T2DM. The maintenance of glucose homeostasis is a complex physiological process that is dependent on both hepatic gluconeogenesis and glycogenolysis. During fasting periods, gluconeogenesis, a process regulated by glucagon, becomes particularly crucial (Consoli, 1992; Magnusson et al., 1992; Wajngot et al., 2001; Boden, 2004). In the context of T2DM, dysregulated glucagon secretion can lead to an overactivation of gluconeogenesis, and consequently, an increase in fasting plasma glucose concentrations. Therefore, therapeutic approaches that target the reduction of glucagon levels or inhibiting its downstream effects are of paramount importance in the management of hyperglycemia in individuals with T2DM.

Hepatic gluconeogenesis is intricately regulated by a variety of factors, including substrate levels, metabolites, and hormonal influences (Zhang X. et al., 2019; Sharabi et al., 2019). Amino acids and free fatty acids, derived from extrahepatic sources, can modulate this process both directly and indirectly (Mc and Gi, 2018; Pan et al., 2022). During periods of starvation, amino acids released from protein breakdown serve as key substrates for hepatic gluconeogenesis. The hepatic response to glucagon is predominantly mediated through the cyclic AMP (cAMP)/protein kinase A (PKA) signaling pathway (Gonzalez and Montminy, 1989). Glucagon plays a pivotal role in the interplay between amino acid metabolism and the urea cycle within the liver. It facilitates the regulation of amino acid metabolism through its influence on urea production (Hu et al., 2023). In a reciprocal manner, circulating amino acids can also prompt the secretion of glucagon from pancreatic α-cells, establishing a feedback loop (Holst et al., 2017; Wewer Albrechtsen et al., 2019). This interdependence underscores the significant role of amino acid metabolism in maintaining hepatic glucose homeostasis. The dynamic interaction between glucagon and amino acid levels is crucial for the liver’s ability to balance glucose production and utilization, ensuring a stable internal glucose environment.

The urea cycle, alternatively referred to as the ornithine cycle, is a critical metabolic pathway responsible for the detoxification and elimination of ammonia generated from the metabolism of amino acids within the body (Schimke, 1962; Gropman et al., 2007). This cycle plays a vital role in the removal of excess nitrogen and ammonia that result from the breakdown of proteins or the synthesis of nitrogenous compounds in humans. Carbamoyl phosphate synthetase 1 (CPS1) is the first and a rate-limiting enzyme in the urea cycle, which catalyzes the condensation of ammonia with bicarbonate to produce carbamyl phosphate, a precursor to urea. CPS1 is indispensable in human metabolism, not only marking the entry point of ammonia into the urea cycle, but also having specific contribution to hepatic gluconeogenesis, however, is not well understood and remains an area of ongoing research. Understanding the interplay between CPS1 and gluconeogenesis could provide insights into the broader metabolic processes within the liver and their implications for health and disease.

Currently available therapeutic agents for T2DM have demonstrated remarkable efficacy. Unfortunately, these medications may also be associated with undesired side effects (Upadhyay et al., 2018; Shetty et al., 2022). By utilizing Traditional Chinese Medicine (TCM) syndrome differentiation and selecting appropriate drugs based on specific symptoms, Chinese medicine can effectively maintain lipid and glucose homeostasis while minimizing side effects, thereby preventing further deterioration of diabetes (Zhang et al., 2019b; 2019a; Lou et al., 2019; Wu G. D. et al., 2022). The treatment of diabetes using TCM holds great potential in achieving broad therapeutic outcomes.

This study was designed to elucidate the involvement of CPS1 in metabolic disorders, with a particular focus on its role in hepatic gluconeogenesis. Our findings indicated that CPS1 exerts its regulatory effect on hepatic gluconeogenesis predominantly through the Ca^2+^/CaMKII/FOXO1 signaling cascade. Furthermore, the research revealed that cynarin, a compound derived from the artichoke plant, had the capacity to attenuate the hepatic glucagon response in a CPS1-dependent manner. This discovery suggested that CPS1 may serve as a novel target for therapeutic interventions aimed at modulating gluconeogenesis and addressing metabolic dysregulations associated with conditions such as diabetes.

## 2 Materials and methods

### 2.1 Animals

For long-term model, C57BL/6J male mice were fed rodent chow diet (10% kcal from fat; Xietong Organism, China) or switched to a high-fat diet (HFD, 60% kcal from fat; D12492; Research diet, America) at 8 weeks of age and fed for 12 weeks. Body weights and food intake were recorded twice a week. For glucagon model, C57BL/6J male mice were injected with 2 mg/kg glucagon intraperitoneally. Mice were housed with a 12-h/12-h light-dark cycle with free access to food and water. All animal treatments were approved by the Animal Ethics Committee of China Pharmaceutical University (protocol no. 2020-12-009). Metformin (200 mg/kg) or cynarin (50, 100 mg/kg) were given by gavage.

### 2.2 AAV8-mediated gene expression and knockdown

AAV8-CPS1 shRNA were injected to generate hepatocyte-specific CPS1 knockdown mice or AAV8- NC for control mice. 6 to 8-week-old mice were intraventricularly injected with AAV8 using a 29-gauge insulin syringe (BD). All tests were performed 4–5 weeks after AAV injection. The shRNA oligo used in our experiments are as follows:

NC shRNA: TTC​TCC​GAA​CGT​GTC​ACG​T; CPS1 shRNA: GCU​CUU​GCA​CAG​CCA​CUA​AUU.

### 2.3 Glucagon, pyruvate and oral glucose tolerance tests

For glucagon tolerance tests (GTT), mice were fasted for 12 h before i.p. injections of 2 mg/kg glucagon in saline solution. For oral glucose tolerance tests (OGTT), mice were fasted for 12 h before administered glucose intragastrically (i.g.) at a dosage of 2.5 g/kg body weight. For pyruvate tolerance tests (PTT), mice were fasted for 12 h before i.p. injections of 2 mg/kg glucagon in saline solution. Blood glucose levels were measured at 0, 15, 30, 60, 90, and 120 min after the injection.

### 2.4 Hepatocyte culture and transfection

Isolated primary hepatocytes were cultured in the Dulbecco’s modified eagle medium (DMEM) with 10% (v/v) fetal bovine serum (FBS). Cells were transfected with CPS1 siRNA using Lipofectamine^®^ 2000 transfection reagent (ThermoFisher, America) at 70% confluence. Negative control siRNA was used as a control. CPS1 plasmid were also used to promote the expression of CPS1 while pcDNA3.1 were used as a control.

NC siRNA: UUC​UCC​GAA​CGU​GUC​ACG​UTT

ACG​UGA​CAC​GUU​CGG​AGA​ATT;

CPS1 siRNA: GCU​CUU​GCA​CAG​CCA​CUA​AUU

AAU​UAG​UGG​CUG​UGC​AAG​AGC.

### 2.5 Quantitative PCR

Total RNA was extracted from cells and tissue samples using High Pure RNA Isolation Kit (Vazyme, China). Equal amounts of RNA samples were used for cDNA synthesis with a HIFair^®^ Ш 1st Strand cDNA Synthesis SuperMix for qPCR (gDNA digester plus) (Yeasen, China). Quantitative PCR analysis was carried out using a Hieff^®^ qPCR SYBR Green Master Mix (Low Rox Plus) (Yeasen, China). The primers used for quantitative PCR are listed in Table 1.

**TABLE 1:** Primer pairs for qPCR.

Genes	Sense (5′-3′)	Anti-sense (5′-3′)
*G6pc* (mice)	CGA​CTC​GCT​ATC​TCC​AAG​TGA	GTT​GAA​CCA​GTC​TCC​GAC​CA
*Pck1* (mice)	AAG​CAT​TCA​ACG​CCA​GGT​TC	GGG​CGA​GTC​TGT​CAG​TTC​AAT
*Pgc1a* (mice)	TAT​GGA​GTG​ACA​TAG​AGT​GTG​CT	GTC​GCT​ACA​CCA​CTT​CAA​TCC
*Actb* (mice)	AGT​GTG​ACG​TTG​ACA​TCC​GTA	GCC​AGA​GCA​GTA​ATC​TCC​TTC​T
*Cps1* (mice)	ACA​TGG​TGA​CCA​AGA​TTC​CTC​G	TTC​CTC​AAA​GGT​GCG​ACC​AAT

### 2.6 Western blotting

The total protein concentration was quantified with a BCA assay kit (Beyotime, China) for the normalization of assayed samples. The samples were applied to 8%–12% SDS-PAGE gels and probed with antibodies. Antibodies are listed in Table 2. Band intensity was measured using ImageJ software.

**TABLE 2:** Primary antibodies for Western blotting.

Antibody	Catalog	Company	Application	Dilution
CPS1	Ab129076	Abcam	WB	1:1,000
β-actin	bs-00612R	Bioss	WB	1:1,000
phospho-PKA substrates	9621S	CST	WB	1:1,000
FOXO1	2880S	CST	WB/IF	1:1,000/1:200
p-CaMKII	AF3493	Affinity	WB	1:1,000
CaMKII	WL03453	Wanlei	WB	1:1,000
Mouse anti-rabbit IgG	ZB-2301	Zhongshanjinqiao	WB	1:1,000
Goat anti-mouse IgG	ZB-2305	Zhongshanjinqiao	WB	1:1,000

### 2.7 Hepatic glucose production

After fast the cells for 2 h in Krebs-Ringer HEPES (KRH) buffer, primary hepatocytes were washed by PBS and incubated in KRH buffer supplemented with 10 mM pyruvate, 100 nM glucagon for 6 h. The cell supernatant was collected for glucose analysis using the Glucose Assay Kit (Jiancheng bioengineering, China), and normalized to total cellular protein content.

### 2.8 Immunofluorescence

For fluorescence visualization of antibody reactions, primary antibodies were detected using secondary antibodies labeled with the fluorochromes FITC (Beyotime, China). To detect cell nuclei, primary hepatocytes were fluoresced with DAPI (Beyotime, China). Confocal images were acquired using a confocal scanning microscope (Olympus, Japan). All images were acquired with an 63× oil (NA 1.42) immersion objective lenses.

### 2.9 Molecular docking

We apply Surflex-Dock, a submodule of SYBYL software, for virtual screening. Download the three-dimensional structure of CPS1 protein from the protein database PDB.org and hydrogenate CPS1 protein. Select the active natural products library containing 724 compounds from www.MedChenExpress.com, download the three-dimensional structure of natural product molecules, and hydrotreat them. The structure optimization of the compounds should be made in advance. The docking process: 1) Generate ligand fragments to reduce the image construction space; 2) Superimpose the ligand fragment onto the prototype probe; 3) The ligand fragment for docking the remaining part. The docking results will be analyzed.

### 2.10 Statistical analysis

Data analysis was carried out by software GraphPad Prism 9. Data were expressed as mean ± standard error of the mean (SEM). Statistical analysis of difference between two groups was performed using a two-tailed Student’s t-test, and comparison for multiple groups was performed by one-way ANOVA. *p* values of less than 0.05 and 0.01 were to be considered significant and very significant, respectively, and they were expressed as *p < 0.05, **p < 0.01, ***p < 0.001.

## 3 Results

### 3.1 CPS1 activates glucagon-induced hepatic gluconeogenesis

**FIGURE 1:**
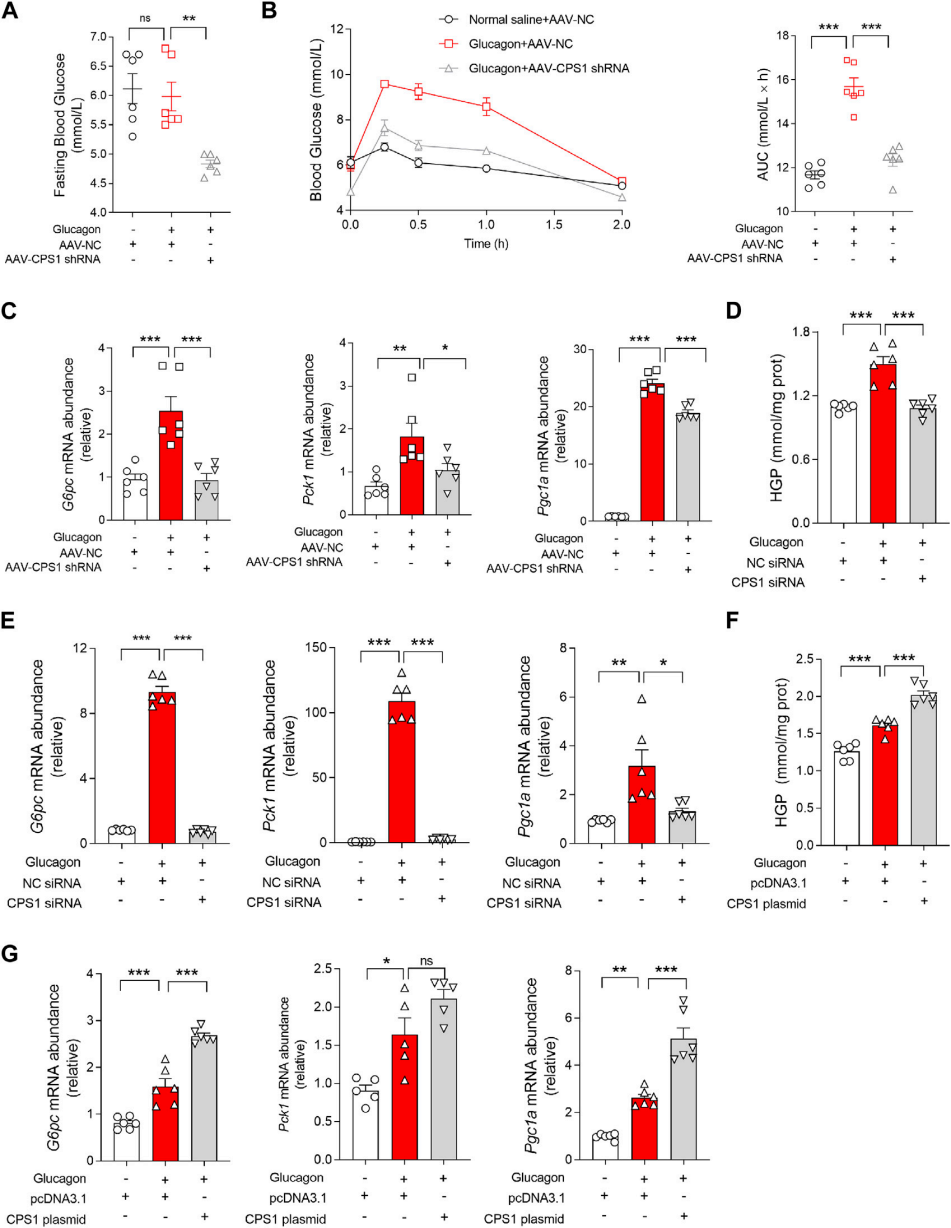
CPS1 activated glucagon-induced gluconeogenesis. **(A)** Fasting blood glucose of CPS1 liver specific knockdown mice (*n* = 6). **(B)** Blood glucose levels and AUC for mice treated with AAV8-CPS1 shRNA or AAV8-NC with glucagon challenge (2 mg/kg, *n* = 6). **(C)** qPCR analysis of the expression of *G6pc*, *Pck1*, and *Pgc1a* in the liver from the mice in panel **(B)** (*n* = 6). **(D)** Hepatic glucose production treated with CPS1 siRNA or NC siRNA stimulated by glucagon (100 nM, 1 h) in hepatocytes (*n* = 6). **(E)** qPCR analysis of the expression of *G6pc*, *Pck1*, and *Pgc1a* in panel **(D)** (*n* = 6). **(F)** Hepatic glucose production treated with CPS1 plasmid or pcDNA3.1 stimulated by glucagon (100 nM, 1 h) in hepatocytes (*n* = 6). **(G)** qPCR analysis of the expression of *G6pc*, *Pck1*, and *Pgc1a* in panel **(F)** (*n* = 6). AUC, area under the curve; HGP, hepatic glucose production; AAV, adeno-associated virus. Data were analyzed by one-way ANOVA. All values are represented as mean ± SEM. *p < 0.05, **p < 0.01, ***p < 0.001.

To explore the role of CPS1 in hepatic gluconeogenesis, we utilized adeno-associated virus serotype 8 (AAV8) carrying small hairpin RNA (shRNA) to generate mice with liver-specific knockdown of CPS1 (Supplementary Figure S1). In the glucagon tolerance test, we observed that the hepatic knockdown of CPS1 notably reduced fasting blood glucose levels (Figure 1A) and subsequently weaked the glucagon-induced hyperglycemia in mice (Figure 1B). Furthermore, the expression levels of key hepatic gluconeogenic genes, including *Pck1*, *G6pc*, and *Pgc1a*, were markedly decreased in CPS1 knockdown mice when subjected to a glucagon challenge (Figure 1C).

Similarly, *in vitro* studies using primary hepatocytes mirrored the *in vivo* findings. CPS1 knockdown (Supplementary Figure S2) effectively counteracted the glucagon-induced expression of hepatic glucose production (Figure 1D) and hepatic gluconeogenic genes, including *G6pc* and *Pck1* (Figure 1E). In contrast, overexpression of CPS1 (Supplementary Figure S3) significantly enhanced glucagon-stimulated hepatic glucose production (Figure 1F) and gluconeogenic genes such as *G6pc* and *Pgc1a* (Figure 1G). These results underscored the pivotal role of CPS1 in the regulation of hepatic gluconeogenesis and its responsiveness to glucagon signaling.

### 3.2 CPS1 knockdown attenuates hepatic gluconeogenesis disorders in HFD-fed mice

**FIGURE 2:**
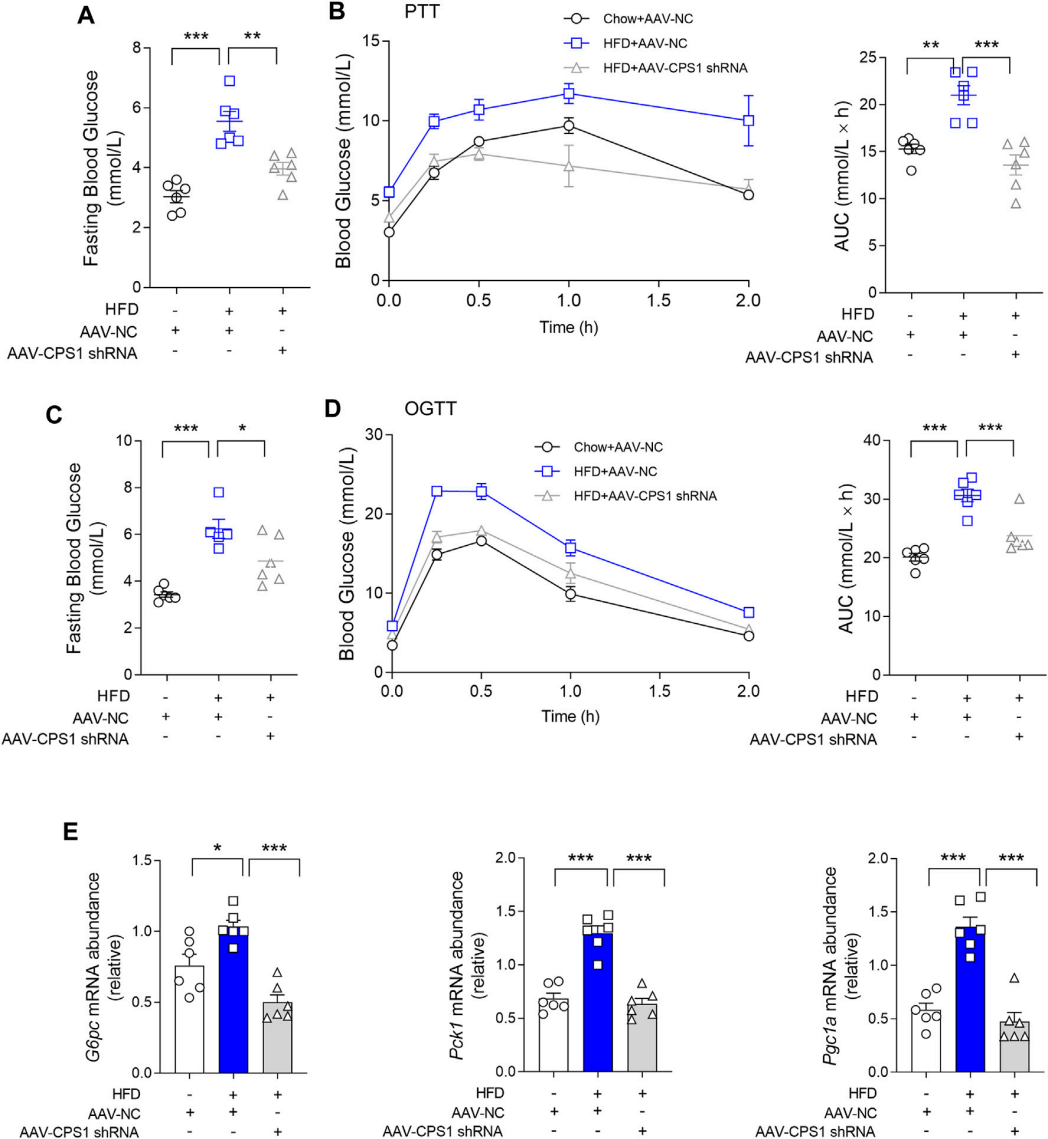
Liver specific knockdown of CPS1 attenuated hepatic gluconeogenesis in HFD-fed mice. **(A)** Fasting blood glucose of CPS1 liver specific knockdown mice after 11 weeks HFD feeding (*n* = 6). **(B)** Pyruvate tolerance test (2 g/kg body weight) after 11 weeks HFD feeding in mice treated with AAV8-CPS1 shRNA or AAV8-NC (*n* = 6). **(C)** Fasting blood glucose of CPS1 knockdown mice after 12 weeks HFD feeding (*n* = 6). **(D)** Oral glucose tolerance test (2.5 g/kg) after 12 weeks HFD feeding in mice treated with AAV8-CPS1 shRNA or AAV8-NC (*n* = 6). **(E)** qPCR analysis of the expression of *G6pc*, *Pck1*, and *Pgc1a* in the liver from the mice in panel **(D)** (*n* = 6). HFD, high-fat diet; PTT, pyruvate tolerance tests; OGTT, oral glucose tolerance tests; AUC, area under the curve; AAV, adeno-associated virus. Data were analyzed by one-way ANOVA. All values are represented as mean ± SEM. *p < 0.05, **p < 0.01, ***p < 0.001.

To further investigate the role of CPS1 in hyperglycemia *in vivo*, hepatic CPS1 was knocked down by tail vein injection of AAV8-shRNA in HFD-fed mice (Supplementary Figure S4A). HFD feeding resulted in fasting hyperglycemia at weeks 10 and 12 (Figures 2A, C) and impaired pyruvate and oral glucose tolerance in mice (Figures 2B, D). Our findings revealed that the knockdown of CPS1 effectively reduced fasting blood glucose levels (Figures 2A, C) and ameliorated both pyruvate tolerance (Figure 2B) and oral glucose tolerance (Figure 2D). In line with these metabolic improvements, the hepatic expression of gluconeogenic genes *Pck1*, *G6pc*, and *Pgc1a* were significantly downregulated in HFD-fed mice following CPS1 knockdown (Figure 2E).

Of note, CPS1 knockdown also correlated with a decrease in body weight gain and reduced fat mass. Additionally, we observed a notable reduction in lipid accumulation within the liver of mice maintained on HFD feeding (Supplementary Figures S4B, D–E). The experimental outcomes demonstrated that the knockdown of CPS1 in liver resulted in a reduction of abdominal adipose tissue accumulation (Supplementary Figure S4B). Concurrently, this intervention was associated with a notable reduction in overall body weight (Supplementary Figure S4D). Oil Red O staining indicated a significant reduction in lipid deposition within the hepatic tissue (Supplementary Figure S4E). It is important to note that these beneficial effects were achieved without any significant alteration in food intake (Supplementary Figure S4C). These results highlighted the potential therapeutic implications of CPS1 modulation in the context of diet-induced metabolic disorders.

### 3.3 CPS1 promotes Ca^2+^ release to modulate hepatic gluconeogenesis

The hepatic response to glucagon is predominantly orchestrated through the activation of the cAMP/PKA signaling pathway. However, knockdown of CPS1 in hepatocytes did not modulate the expression levels of PKA (Supplementary Figure S5A). Correspondingly, the PKA inhibitor H-89 did not participate in the gluconeogenesis regulatory process governed by CPS1 (Supplementary Figures S5B–C). These findings suggested that the regulatory influence of CPS1 on gluconeogenesis bypasses the canonical pathway.

**FIGURE 3:**
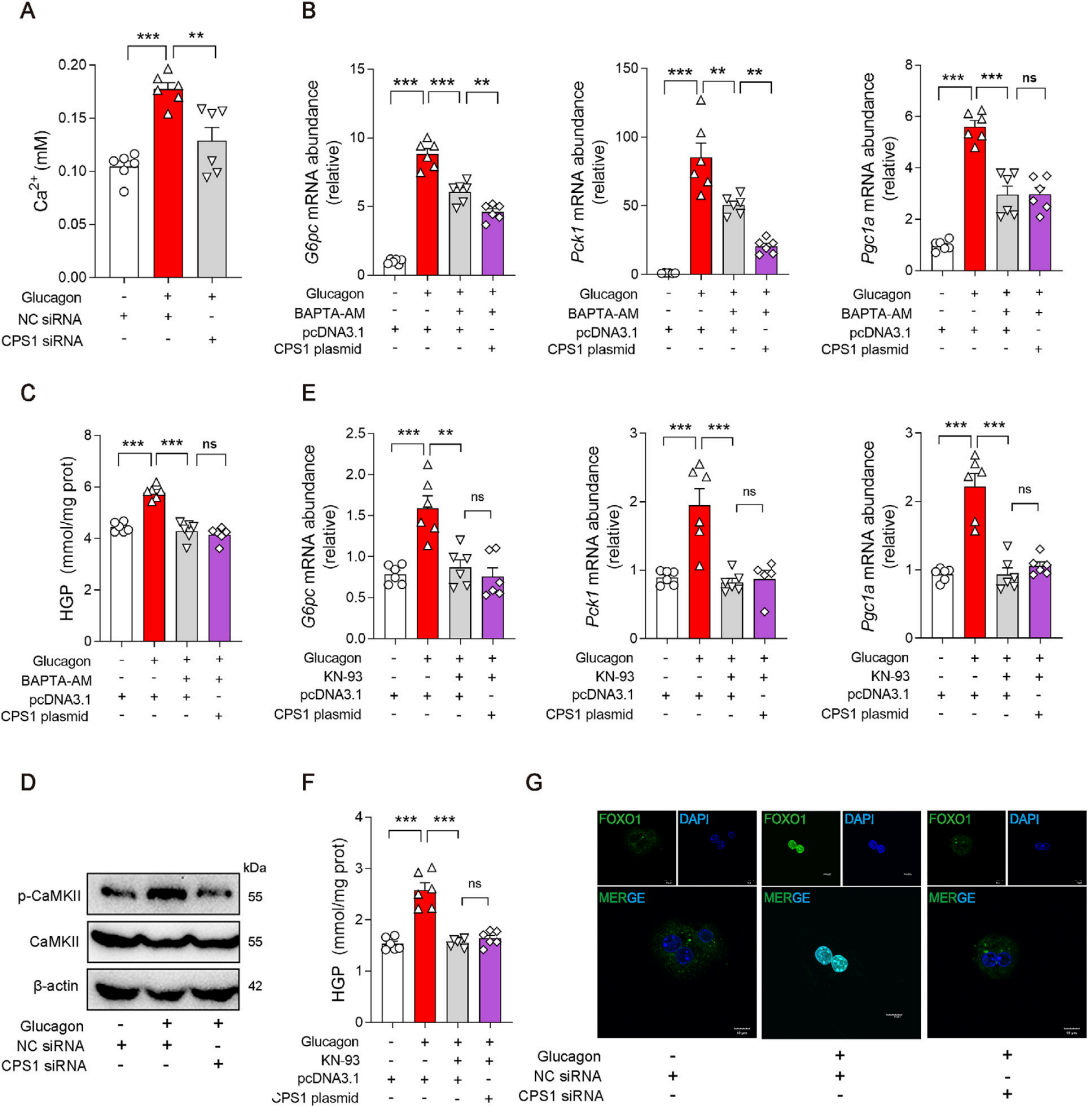
CPS1 activated CaMKII/FOXO1 pathway promoted by Ca^2+^ release in glucagon-stimulated hepatocytes. **(A)** Ca^2+^ level in hepatocytes treated with CPS1 siRNA or NC siRNA stimulated by glucagon (100 nM, 1 h) (*n* = 6). **(B)** qPCR analysis of the expression of *G6pc*, *Pck1*, and *Pgc1a* transfected with CPS1 plasmid stimulated by glucagon (100 nM, 1 h) after pretreated with BAPTA-AM (5 μM) for 1 h (*n* = 6). **(C)** Hepatic glucose production in panel **(B)** (*n* = 6). **(D)** Protein levels of CaMKII and p-CaMKII treated with CPS1 siRNA or NC siRNA stimulated by glucagon (100 nM, 1 h) (*n* = 3). **(E)** qPCR analysis of the expression of *G6pc*, *Pck1*, and *Pgc1a* transfected with CPS1 plasmid stimulated by glucagon (100 nM, 1 h) with KN-93 (10 μM, 1 h) (*n* = 6). **(F)** Hepatic glucose production in panel **(E)** (*n* = 6). **(G)** Confocal images of FOXO1 nuclear translocation in panel **(D)**. Scale bars, 10 μm. HGP, hepatic glucose production. Data were analyzed by one-way ANOVA. All values are represented as mean ± SEM. **p < 0.01, ***p < 0.001.

Glucagon also promotes gluconeogenesis by activating specific calcium ion channels or releasing calcium ions from the endoplasmic reticulum. Interestingly, the release of Ca^2+^ in glucagon-stimulated hepatocyte was abrogated by CPS1 knockdown (Figure 3A), suggesting CPS1 as a Ca^2+^ regulator in glucagon response. Calcium chelating agent BAPTA-AM was subsequently used to detect the effect of Ca^2+^ in CPS1 initiated gluconeogenesis. Obviously, the effect of CPS1 on initiating gluconeogenesis was abrogated by BAPTA-AM (Figures 3B, C), indicating that CPS1 could promote gluconeogenesis by upregulating calcium. Inositol 1,4,5-trisphosphate receptor (IP3R) is a Ca^2+^-release channel and plays a crucial role in intracellular calcium signaling. In response to glucagon, IP3 binds to IP3R, leading to the release of Ca^2+^ from the endoplasmic reticulum into the cytosol. IP3R inhibitor XesC was subsequently used to determine the action of IP3R in CPS1-mediated gluconeogenesis. Notedly, the augment of gluconeogenesis by CPS1 overexpression was not affected by XesC (Supplementary Figures S6A, B). Thus, CPS1 regulated hepatic gluconeogenesis is not mediated by IP3R.

### 3.4 CPS1 augment glucagon response by CaMKII/FOXO1 pathway

Calcium-mediated CaMKII/FOXO1 axis plays a significant role in the regulation of gluconeogenesis. In response to glucagon, CaMKII is activated and delivered FOXO1 into the nucleus, where it transcriptionally promotes gene induction of *Pck1* and *G6pc* in cooperation with the co-activator *Pgc1a*. Based on this, we further investigated whether CPS1 mediated hepatic gluconeogenesis through CaMKII/FOXO1 pathway. Of note, knockdown of CPS1 significantly diminished the phosphorylation of CaMKII in response to glucagon stimulation (Figure 3D). KN-93, a specific inhibitor of CaMKII, significantly attenuates the phosphorylation of CaMKII. Overexpression of CPS1 did not affect the inhibitory effect of KN-93 on gluconeogenesis (Figures 3E, F). This suggested that the regulatory role of CPS1 in hepatic gluconeogenesis is dependent on CaMKII. The phosphorylation status of FOXO1 is a critical determinant of its nuclear exclusion. CPS1 knockdown elevated the phosphorylation of FOXO1. Furthermore, immunofluorescence imaging provided evidence that CPS1 knockdown favors the nuclear exclusion of FOXO1 (Figure 3G). Summarily, we proposed that CPS1 modulated hepatic gluconeogenesis by impinging upon the CaMKII/FOXO1 signaling cascade through the regulation of calcium ion release.

### 3.5 *In silico* screening reveals cynarin as a natural CPS1 inhibitor

**FIGURE 4:**
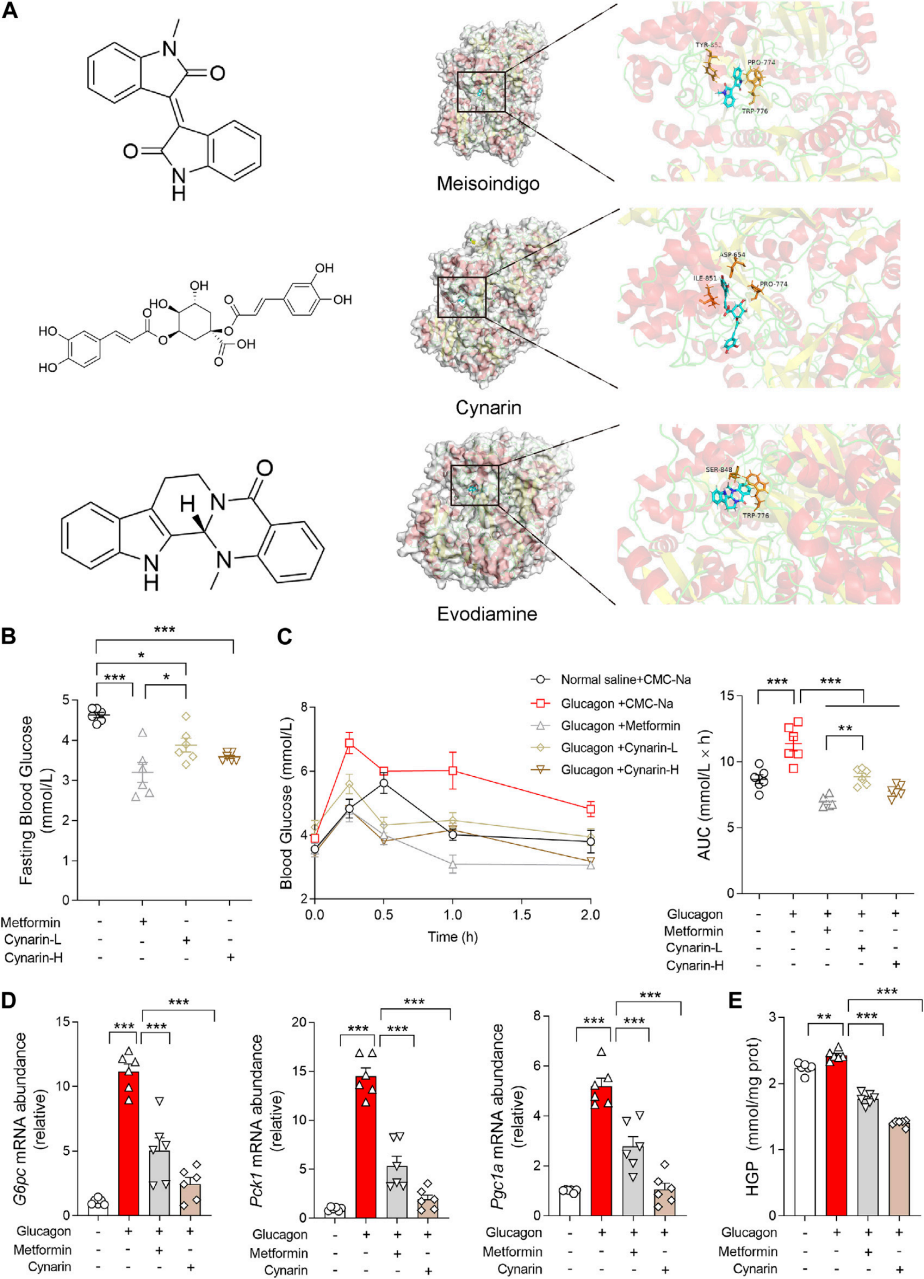
Gluconeogenesis inhibition effects of compounds screened binding to CPS1 by molecular docking. **(A)** Chemical structure formula of meisoindigo, cynarin, evodiamine and prediction of binding sites with CPS1 crystal structure. **(B)** Fasting blood glucose of mice pretreated with cynarin (L: 50 mg/kg; H: 100 mg/kg, 1 h) or metformin (200 mg/kg, 1 h) in mice (*n* = 6). **(C)** Blood glucose levels and AUC for mice pretreated with cynarin (L: 50 mg/kg; H: 100 mg/kg, 1 h) or metformin (200 mg/kg, 1 h) with glucagon challenge (2 mg/kg, *n* = 6). **(D)** qPCR analysis of the expression of *G6pc*, *Pck1*, and *Pgc1a* pretreated with metformin (1 mM, 4 h) or cynarin (20 μM, 4 h), 100 nM glucagon stimulation for 1 h in hepatocytes (*n* = 6). **(E)** Hepatic glucose production in panel **(D)** (*n* = 6). AUC, area under the curve; L, Low concentration; H, High concentration. Data were analyzed by one-way ANOVA. All values are represented as mean ± SEM. *p < 0.05, **p < 0.01, ***p < 0.001.

Given the pivotal role of CPS1 in the regulation of hepatic gluconeogenesis, pharmacological CPS1 inhibition is a potential therapeutic approach for modulating hepatic gluconeogenesis. To this end, an *in silico* molecular docking analysis was conducted utilizing a comprehensive library of natural compounds. Out of 724 natural compounds assessed, we have identified and highlighted the top 10 natural products with the highest binding affinity scores for CPS1 (Table 3). The predicted binding interactions between the top three natural products and the crystal structure of CPS1 were visually represented in Figure 4A, with their corresponding chemical structure formulas displayed in Figure 4A.

**TABLE 3:** Ranking of natural products binding with CPS1.

Catalog_No.	Name	Docking score	Glide gscore	Glide emodel
HY-13680	Meisoindigo	−11.479	−11.479	−75.095
HY-N0359	Cynarin	−10.783	−10.783	−83.779
HY-N0114	Evodiamine	−10.712	−10.712	−67.671
HY-N0058	4,5-Dicaffeoylquinic acid	−10.685	−10.685	−89.305
HY-N0261	Aurantio-obtusin	−10.650	−10.691	−84.624
HY-N0134	Tanshinone I	−10.644	−10.644	−73.372
HY-13065	Isobavachalcone	−10.576	−10.623	−79.175
HY-N0120A	Polydatin	−10.562	−10.562	−87.920
HY-B1756	Rotenone	−10.454	−10.454	−75.489

Of particular interest, cynarin, a bioactive compound derived from artichoke, was found to effectively reduce the fasting blood glucose that was upregulated by glucagon (Figures 4B, C). Specifically, we have documented the inhibitory of cynarin on gluconeogenesis, findings were further substantiated through *in vitro* experimentation. The data evinced that both cynarin and metformin exert ameliorative effects on gluconeogenesis, with cynarin demonstrating a superior efficacy in comparison to metformin (Figure 4D). This observation was congruent with the outcomes of our hepatic glucose production assays (Figure 4E). In light of these findings, we posited that cynarin may serve as a hypoglycemic agent by specifically targeting and modulating CPS1 activity.

### 3.6 Cynarin suppressed hepatic glucagon response via CPS1/CaMKII/FOXO1 pathway

**FIGURE 5:**
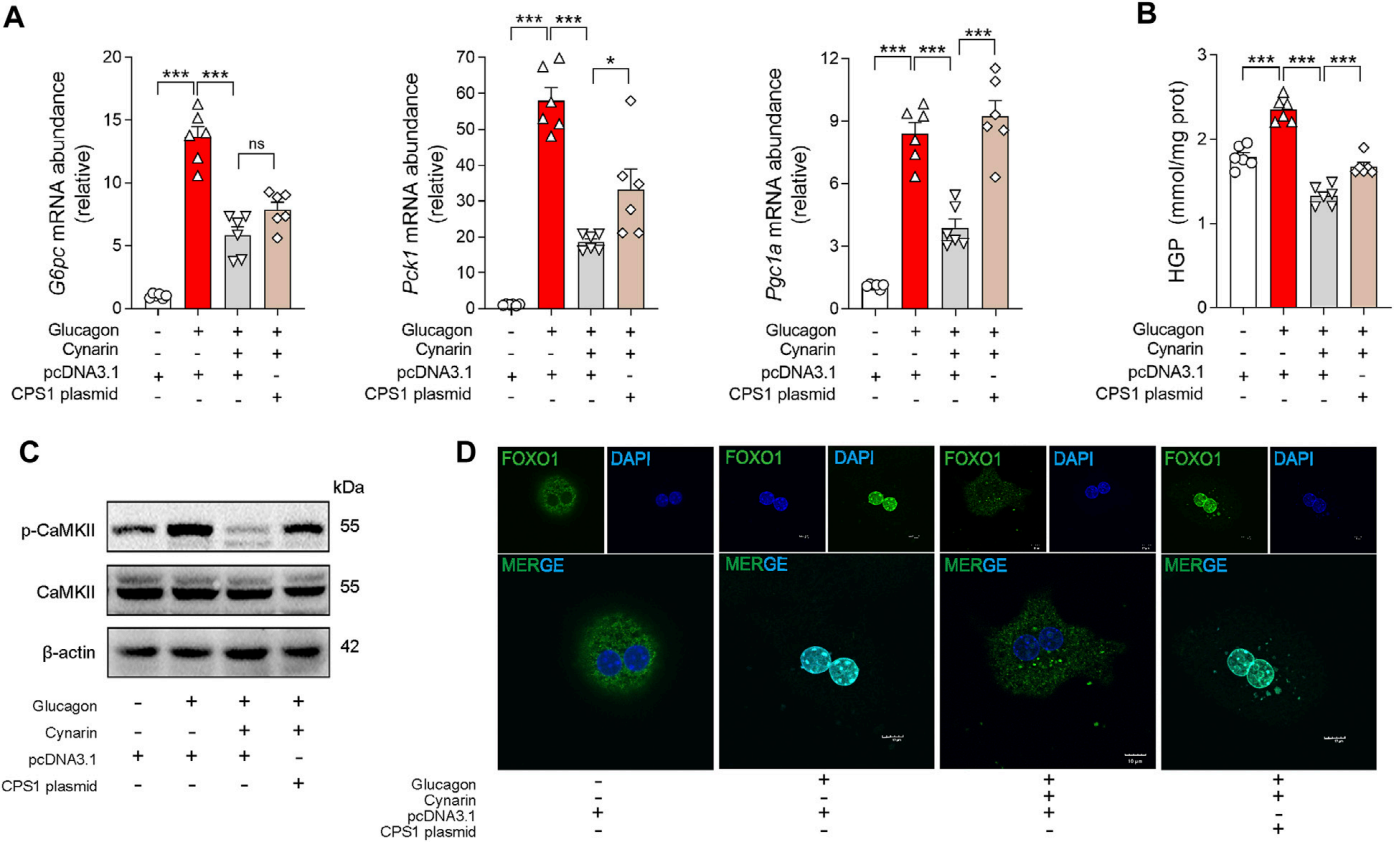
Cynarin suppressed CPS1/CaMKII/FOXO1 pathway to diminish hepatic glucagon response in hepatocytes. **(A)** qPCR analysis of the expression of *G6pc*, *Pck1*, and *Pgc1a* transfected with CPS1 plasmid or pcDNA3.1 with cynarin (20 μM, 4 h) stimulated by glucagon (100 nM, 1 h) (*n* = 6). **(B)** Hepatic glucose production in panel **(A)** (*n* = 6). **(C)** Protein levels of CAMKII and p-CaMKⅡ in panel **(A)** (*n* = 3). **(D)** Confocal images of FOXO1 nuclear translocation in panel **(A)**. Scale bars, 10 μm. HGP, hepatic glucose production. Data were analyzed by one-way ANOVA. All values are represented as mean ± SEM. *p < 0.05, ***p < 0.001.

To elucidate the mechanisms of cynarin in preventing gluconeogenesis, primary hepatocytes were transfected with CPS1 plasmid. The hypoglycemic influence of cynarin was significantly attenuated in hepatocytes with CPS1 overexpression (Figures 5A, B). This observation underscored the crucial role of CPS1 in mediating the hepatic gluconeogenesis regulatory effects of cynarin.

In addition, we examined the role of cynarin in modulating the CaMKII/FOXO1 pathway. Cynarin markedly elevated the phosphorylation of FOXO1 in the presence of glucagon and concurrently reduced the phosphorylation levels of CaMKII. Intriguingly, this effect was abrogated by CPS1 overexpression in primary hepatocytes (Figure 5C). Furthermore, overexpression of CPS1 hindered the regulatory influence of cynarin on the nuclear translocation of FOXO1 (Figure 5D). These findings suggested that cynarin’s suppressive effect on the hepatic glucagon response is linked to its regulatory actions on the CaMKII/FOXO1 pathway, with CPS1 playing an indispensable role.

## 4 Discussion

**FIGURE 6:**
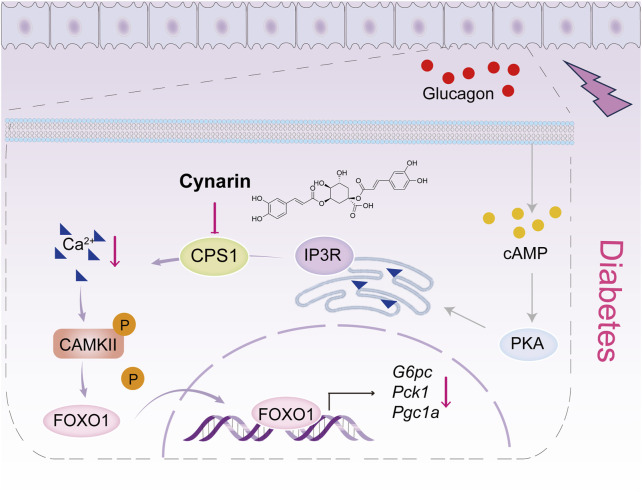
Scheme of the role for CPS1 in activating Ca^2+^/CaMKII/FOXO1 signaling cascade to enhance hepatic gluconeogenesis. The CaMKII pathway is positioned downstream of the cAMP/PKA signaling cascade. Under normal conditions, Ca^2+^ are maintained in a steady state, and CaMKII remains inactive. This inactivation prevents the nuclear translocation of the FOXO1 transcription factor by modifying its phosphorylation state, which is crucial for the regulation of gluconeogenic genes and hepatic glucose production. Upon glucagon stimulation, CPS1 triggers the release of Ca^2+^ from the endoplasmic reticulum into the cytosol. This release activates CaMKII, which in turn enhances the transcriptional activity of FOXO1, thereby promoting hepatic gluconeogenesis. The natural compound cynarin targets CPS1 to inhibit this gluconeogenic process.

In this work, we delineated the pivotal role of CPS1 in hepatic gluconeogenesis. Our findings revealed that CPS1, a rate-limiting enzyme in the urea cycle, significantly exacerbates glucagon-induced hyperglycemia. Conversely, the knockdown of CPS1 attenuated the glucagon response. Notably, CPS1 modulated hepatic gluconeogenesis predominantly through the CaMKII/FOXO1 signaling pathway, diverging from the conventional activation of the cAMP/PKA pathway. Additionally, the hypoglycemic effects of cynarin, a compound derived from the artichoke plant, were partially mediated by CPS1 (Figure 6). Collectively, our data suggested a promising therapeutic approach targeting CPS1 as a potential intervention for type 2 diabetes.

Typically, glucagon activates PKA to directly phosphorylate CREB, which in turn transcriptionally induces the PGC-1α, a key coactivator of FOXO1 transcription factor (Herzig et al., 2001). However, in livers overexpressed CPS1, no significant difference in p-CREB levels were observed, suggesting alternative pathways may be at play. Beyond the cAMP/PKA signaling axis, glucagon also engages the calcium/CaMKII pathway, which operates downstream of the cAMP/PKA cascade. CaMKII, a serine/threonine kinase, is activated by cAMP and glucagon in a calcium- and IP3R-dependent manner (Ozcan et al., 2012). This activation is pivotal for mediating the effects of Ca^2+^ (Couchonnal and Anderson, 2008; Singer, 2012). For instance, glucagon and cAMP are known to elevate intracellular Ca^2+^ levels, and the chelation of Ca^2+^ has been demonstrated to attenuate glucagon-induced hepatic gene expression and glucose production (Staddon and Hansford, 1989; Bygrave and Benedetti, 1993; Mine et al., 1993). Previous studies have also correlated intracellular Ca^2+^ with the regulation of gluconeogenesis (Friedmann and Rasmussen, 1970; Kraus-Friedmann and Feng, 1996; Marques-da-Silva et al., 1997). The inhibition of CaMKII has been shown to impede the nuclear translocation of FOXO1 by altering its phosphorylation state, thereby disrupting fasting- or glucagon-induced gluconeogenesis (Ozcan et al., 2012). Similarly, our findings suggested that CPS1 may modulate hepatic gluconeogenesis by influencing the CaMKII/FOXO1 signaling cascade, potentially through the regulation of calcium ion release. While glucagon operates in the liver alongside other pathways to ensure the nuclear localization of an array of transcription factors that mediate hepatic glucose production, the precise mechanism by which CPS1 regulates calcium ions remains to be elucidated and warrants further investigation.

Glucose, fatty acids, and amino acids serve as the primary sources of cellular energy. The breakdown of amino acids can result in the production of toxic ammonia. Detoxification of ammonia through the urea cycle is essential for preventing hyperammonemia and hepatic encephalopathy (Gropman et al., 2007). Glucagon, a hormone secreted by the pancreas, stimulates hepatic amino acid metabolism, particularly ureagenesis. Conversely, glucagon secretion is also sensitive to amino acids, which can stimulate glucagon secretion by increasing the number of alpha cells, thus establishing a reciprocal feedback loop (Holst et al., 2017; Wewer Albrechtsen et al., 2019). It has been documented that the activities of the five key enzymes in the urea cycle are elevated during fasting and in response to glucagon (Schimke, 1962; Husson et al., 1987; Li et al., 2016), predominantly through transcriptional, posttranscriptional, and posttranslational mechanisms (Ulbright and Snodgrass, 1993; Estall et al., 2009). Under the influence of CPS1, ammonia is converted to carbamoyl phosphate, which then reacts with ornithine to form citrulline through the catalytic action of ornithine transcarbamylase. Citrulline subsequently channels ammonia into the urea cycle. CPS1, as the rate-limiting enzyme, controls the influx of substrates under physiological conditions. Overexpression of CPS1 has been shown to exacerbate HFD-induced excessive activation of gluconeogenesis, as well as glucagon-induced gluconeogenesis both *in vivo* and *in vitro*. However, our study did not include the assessment of CPS1 enzyme activity, which could be a critical factor in hepatic gluconeogenesis. Further investigation is necessary, including the use of liver-specific CPS1 knockout mice for *in vivo* experiments to confirm the role of CPS1 in hepatic gluconeogenesis.

Artichoke, scientifically known for its compounds such as cynarin and chlorogenic acid, is a traditional Chinese medicinal herb with a diverse range of pharmacological properties. Studies have suggested that cynarin can be utilized in the treatment of liver damage (Tong et al., 2017), gouty arthritis (Wu C. et al., 2022), endothelial inflammation (Kim et al., 2022) and intervertebral disc degeneration (Zhang et al., 2023). These effects are mediated through the inhibition of inflammatory or pyroptosis. E. Heidarian and colleagues reported that artichoke extract, rich in cynarin, can effectively reduce blood glucose levels (Heidarian and Rafieian-Kopaei, 2013). However, the precise hypoglycemic mechanism of cynarin remains to be fully elucidated. Our research indicated that overexpression of CPS1 negates the inhibitory effect of cynarin on hepatic gluconeogenesis *in vitro*. We discovered that cynarin, acting as an inhibitor of CPS1, mitigates the excessive activation of hepatic gluconeogenesis via the CaMKII/FOXO1 signaling pathway. These findings offer novel insights into the hypoglycemic effects of cynarin.

In conclusion, this study has delineated a previously unappreciated mechanism by which CPS1 modulates glucagon-induced hepatic gluconeogenesis through the CaMKII/FOXO1 signaling cascade and offered a potential natural inhibitor of CPS1, which may show therapeutic benefits in the management of glucose metabolism disorders.

## Data Availability

The raw data supporting the conclusions of this article will be made available by the authors, without undue reservation.
