# Supplementary material for: CPS1 augments hepatic glucagon response through CaMKII/FOXO1 pathway
Source: Front Pharmacol. 2024 Aug 13;15:1437738. doi: 10.3389/fphar.2024.1437738 (PMC11347310; doi:10.3389/fphar.2024.1437738)

***Supplementary Material***

**1 Supplementary Figures**


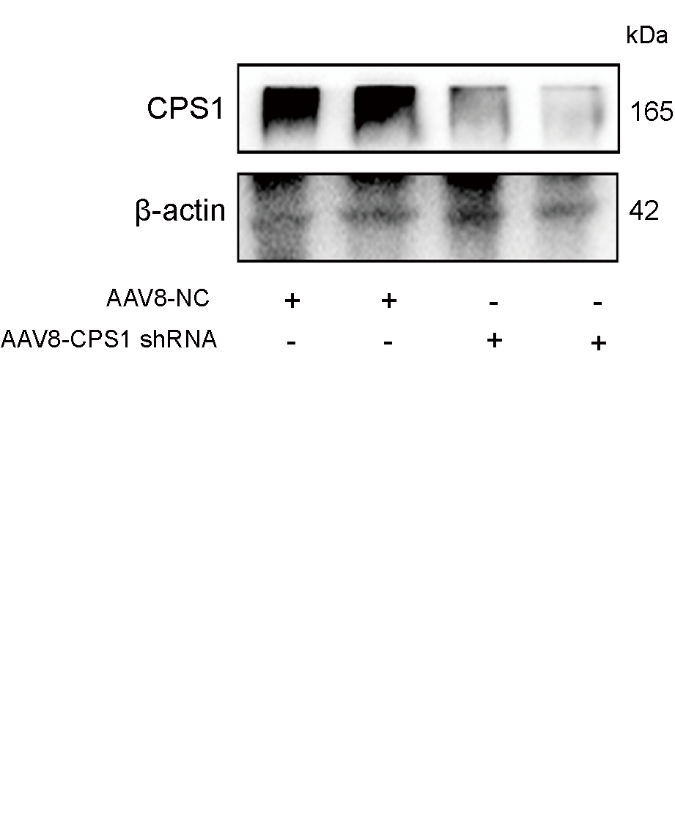


**Supplementary Figure 1 CPS1 was knocked out in the liver of mice.**

CPS1 protein level in the liver of 8-week-old C57BL/6J mice after AAV8-CPS1 shRNA injection (*n* = 2).


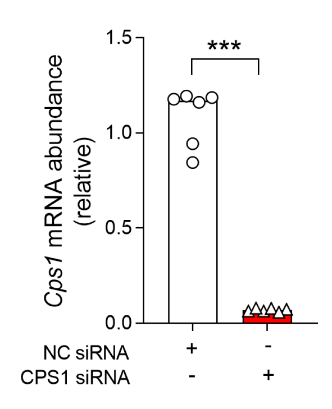


**Supplementary Figure 2 CPS1 was knocked out in hepatocytes.**

mRNA levels of *Cps1* after CPS1 siRNA transfection (*n* = 6). Data were analyzed by two-tailed Student *t*-test. All values are represented as mean ± SEM. ****p* < 0.001.


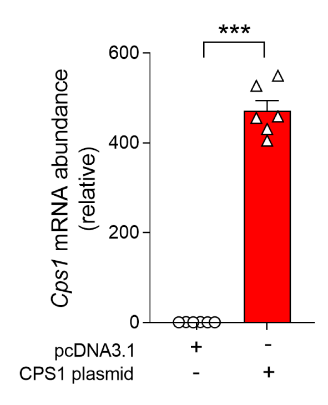


**Supplementary Figure 3 CPS1 was overexpressed in hepatocytes.**

mRNA levels of *Cps1* after CPS1 plasmid transfection (*n* = 6). Data were analyzed by two-tailed Student *t*-test. All values are represented as mean ± SEM. ****p* < 0.001.


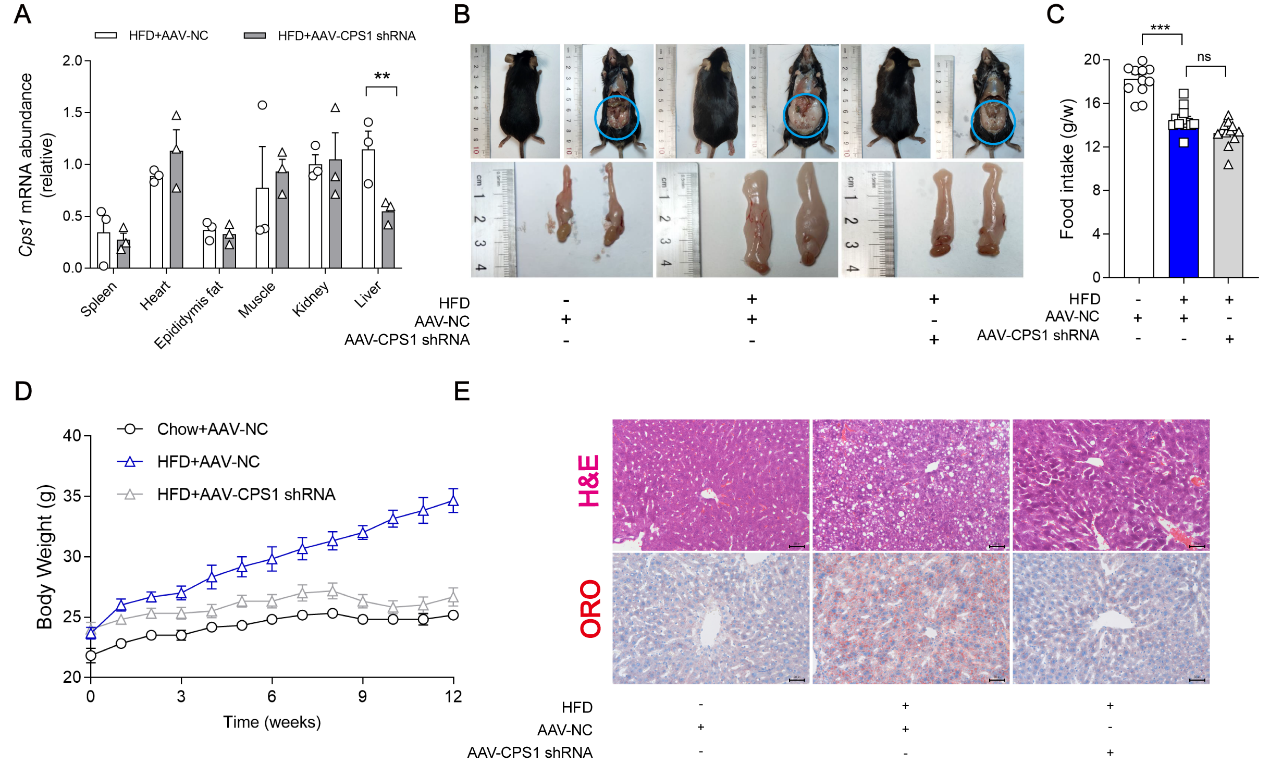


**Supplementary Figure 4** **Liver specific knockdown of CPS1 attenuates hepatic lipid accumulation in HFD-fed mice.**

**(A)** mRNA levels of *Cps1* in different tissues of mice injected with AAV8-CPS1 shRNA or AAV8-NC with HFD feeding (*n* = 3). **(B)** Mouse morphology and fat size of epididymis. Representative images of 3 independent experiments are shown. **(C)** Food intake of mice injected with AAV8-CPS1 shRNA or AAV8-NC fed with chow or HFD (*n* = 12). **(D)** Body weight of mice injected with AAV8-CPS1 shRNA or AAV8-NC fed with NCD or HFD (*n* = 6). **(E)** H&E staining and Oil Red O-stained of liver sections. Scale bars, 50 μm. Representative images of 3 independent experiments are shown. HFD high-fat diet; AAV adeno-associated virus. Data were analyzed by one-way ANOVA. All values are represented as mean ± SEM. ****p* < 0.001.


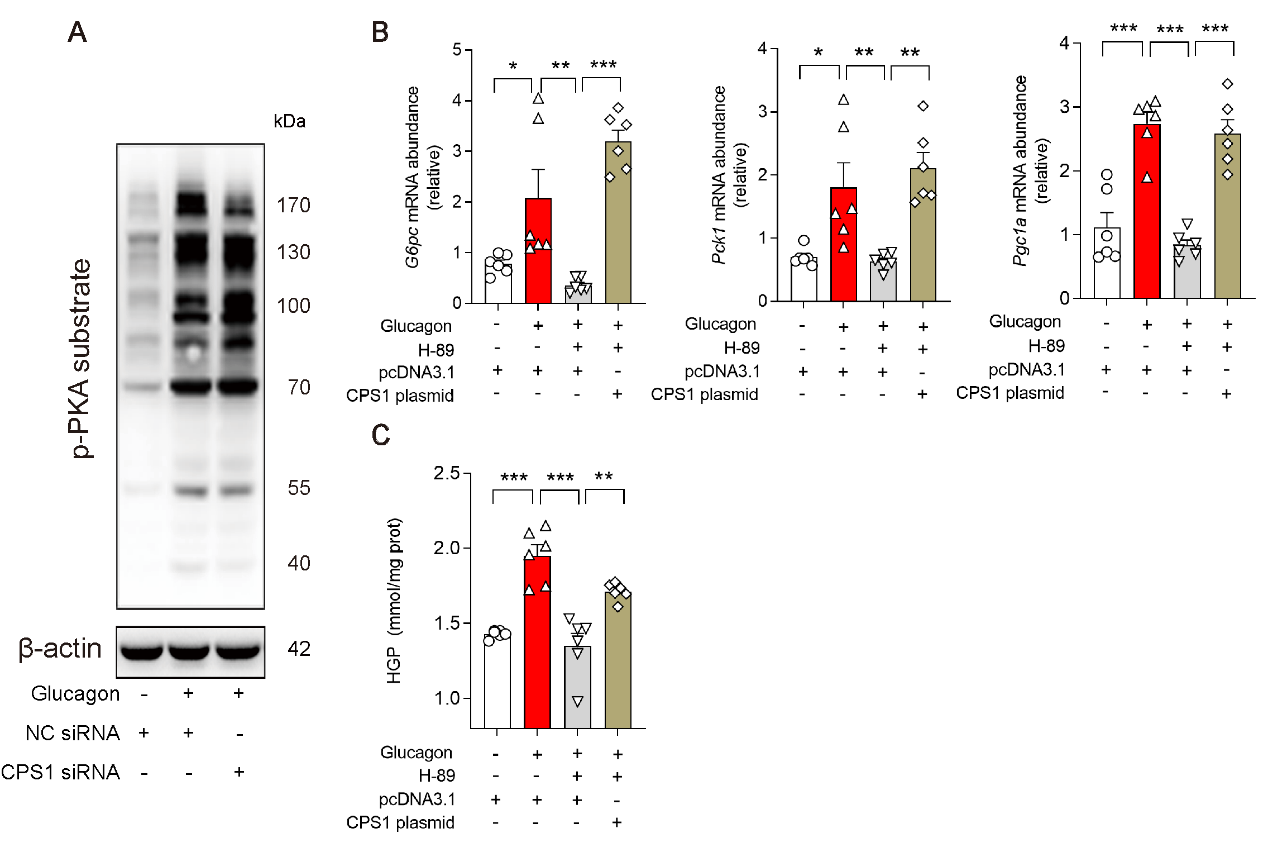


**Supplementary Figure 5 Boost of CPS1 on gluconeogenesis is not regulated by PKA in hepatocytes.**

**(A)** Protein levels of phospho-PKA substrates treated with CPS1 siRNA or NC siRNA stimulated by glucagon (100 nM, 1 h) (*n* = 3). **(B)** qPCR analysis of the expression of *G6pc*, *Pck1*, and *Pgc1a* transfected with CPS1 plasmid stimulated by glucagon (100 nM, 1 h) after pretreated with H89 (200 μM) for 1 h (*n* = 6). **(C)** Hepatic glucose production in panel **B** (*n* = 6). HGP hepatic glucose production. Data were analyzed by one-way ANOVA. All values are represented as mean ± SEM. **p* < 0.05, ***p* < 0.01, ****p* < 0.001.


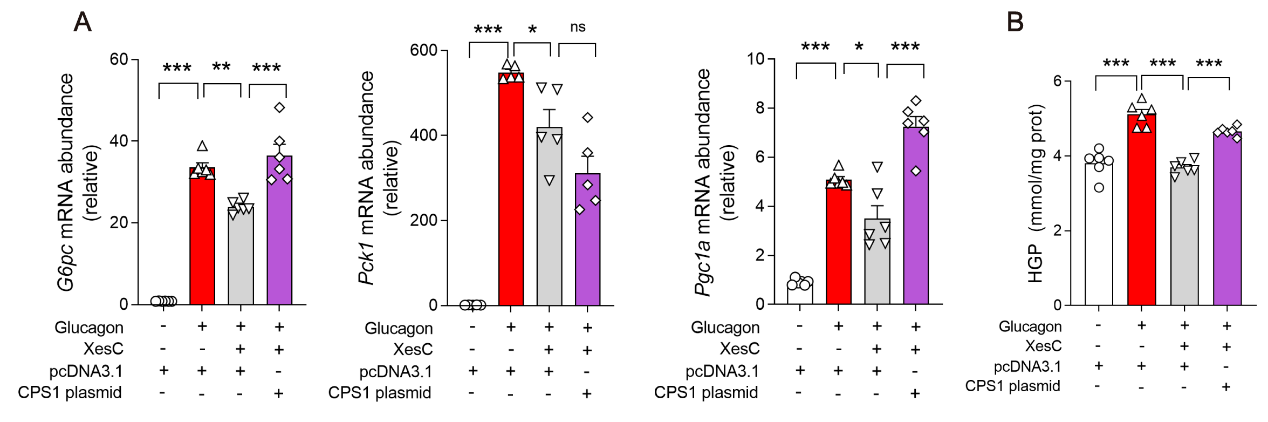


**Supplementary Figure 6 CPS1 promote hepatic gluconeogenesis is not due to IP3R. (A)** qPCR analysis of the expression of *G6pc*, *Pck1*, and *Pgc1a* transfected with CPS1 plasmid stimulated by glucagon (100 nM, 1 h) after pretreated with XesC (0.5 μM) for 1 h (*n* = 6). **(B)** Hepatic glucose production in panel **A** (*n* = 6). HGP hepatic glucose production. Data were analyzed by one-way ANOVA. All values are represented as mean ± SEM. **p* < 0.05, ***p* < 0.01, ****p* < 0.001.


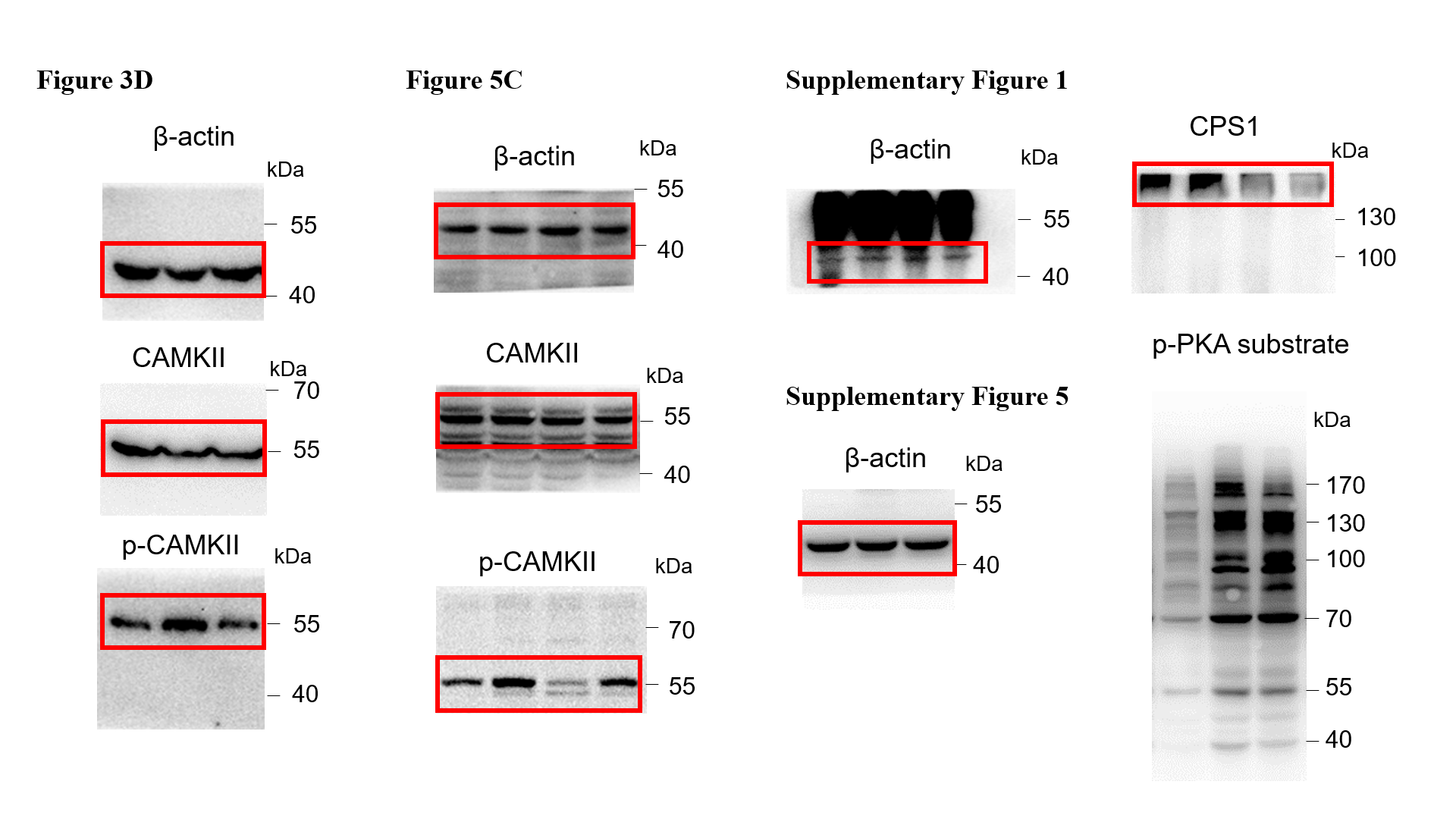

Supplement: Supplementary file 1 [file DataSheet1.docx]
